# Spatio-Temporal Variation in Vegetation Biomass and Its Relationships with Climate Factors in the Xilingol Grasslands, Northern China

**DOI:** 10.1371/journal.pone.0083824

**Published:** 2013-12-16

**Authors:** Tian Gao, Xiuchun Yang, Yunxiang Jin, Hailong Ma, Jinya Li, Haida Yu, Qiangyi Yu, Xiao Zheng, Bin Xu

**Affiliations:** 1 State Key Laboratory of Forest and Soil Ecology, Institute of Applied Ecology, Chinese Academy of Sciences, Shenyang, China; 2 Key Laboratory of Agri-informatics, Ministry of Agriculture / Institute of Agricultural Resources and Regional Planning, Chinese Academy of Agricultural Sciences, Beijing, China; DOE Pacific Northwest National Laboratory, United States of America

## Abstract

Knowledge about grassland biomass and its dynamics is critical for studying regional carbon cycles and for the sustainable use of grassland resources. In this study, we investigated the spatio-temporal variation of biomass in the Xilingol grasslands of northern China. Field-based biomass samples and MODIS time series data sets were used to establish two empirical models based on the relationship of the normalized difference vegetation index (NDVI) with above-ground biomass (AGB) as well as that of AGB with below-ground biomass (BGB). We further explored the climatic controls of these variations. Our results showed that the biomass averaged 99.01 Tg (1 Tg=10^12^ g) over a total area of 19.6×10^4^ km^2^ and fluctuated with no significant trend from 2001 to 2012. The mean biomass density was 505.4 g/m^2^, with 62.6 g/m^2^ in AGB and 442.8 g/m^2^ in BGB, which generally decreased from northeast to southwest and exhibited a large spatial heterogeneity. The year-to-year AGB pattern was generally consistent with the inter-annual variation in the growing season precipitation (GSP), showing a robust positive correlation (R^2^=0.82, P<0.001), but an opposite coupled pattern was observed with the growing season temperature (GST) (R^2^=0.61, P=0.003). Climatic factors also affected the spatial distribution of AGB, which increased progressively with the GSP gradient (R^2^=0.76, P<0.0001) but decreased with an increasing GST (R^2^=0.70, P<0.0001). An improved moisture index that combined the effects of GST and GSP explained more variation in AGB than did precipitation alone (R^2^=0.81, P<0.0001). The relationship between AGB and GSP could be fit by a power function. This increasing slope of the GSP–AGB relationships along the GSP gradient may be partly explained by the GST–GSP spatial pattern in Xilingol. Our findings suggest that the relationships between climatic factors and AGB may be scale-dependent and that multi-scale studies and sufficient long-term field data are needed to examine the relationships between AGB and climatic factors.

## Introduction

Vegetation biomass is an important component of regional carbon cycles, and estimating biomass and its spatio-temporal variation are the basis for studying the carbon (C) cycle [1,2]. Grassland ecosystems have an important role in the regional terrestrial carbon cycle because of their widespread distribution and their high proportion of below-ground biomass [3,4]. In China, grassland is the largest terrestrial ecosystem, occupying approximately 1/3 of the country’s total area, and it plays a key role in animal husbandry in central and western China [5]. In addition, most grassland is distributed in semi-arid and arid zones, which are particularly sensitive to climate change [6]. Therefore, the study of the spatio-temporal variation in grassland biomass and its responses to climate change are critical not only for understanding the potential role of grasslands in the regional terrestrial C cycle but also for the sustainable use of grassland resources [7,8].

A number of studies on grassland biomass have been conducted in China, and *in situ* biomass measurements [9,10] and satellite-based statistical models [11-14] are primarily employed in these investigations. *In situ* biomass measurement is the traditional approach for estimating biomass in grasslands, but it has limitations in terms of both temporal scale and spatial extent [15]. Alternatively, the satellite-based estimation approach combined with corresponding ground-based observations can resolve the scale translation problem to some extent and has thus been widely used to estimate vegetation biomass at the national scale [16]. For example, the changes in grassland biomass in China have been examined during the past two decades using AVHRR-NDVI time series data and ground-based observations [3,11]. However, these studies mostly focused on the spatio-temporal variations of grassland biomass at the national scale. The variations in biomass vegetation may be regionally diverse [17], and their variations within small regions may be ignored at larger scales. Hence, investigations at the regional level are urgently needed to improve our knowledge of the mechanisms underlying biomass variation in grasslands. Additionally, field-measured data are the most basic and direct for biomass estimations in grasslands [9]. However, because field sampling data are limited for years, particularly for below-ground biomass (BGB), biomass assessments at the regional scale are still lacking.

The relationships between biomass and climatic factors have been a key issue in the debate concerning the response of the grassland ecosystem to climatic change [18-23]. In the Eurasian steppe region, precipitation is a principal climate factor impacting grassland ecosystem processes [24] and has thus been frequently used to explain biomass spatial variation [15,25]. Some researchers have reported a positive correlation between precipitation and above-ground biomass (AGB) [24,26-28], but the shape of the precipitation–AGB relationship is still controversial. For example, linear relationships have been reported for the temperate grasslands in Inner Mongolia [29]. However, some recent studies have found that this linearity may be not universal, and exponential relationships have been observed for the temperate grasslands in Inner Mongolia [24,28]. Notably, a recent study suggested that the relationships between vegetation and climatic factors depend on the spatial scale [30]. Previous studies have mostly been conducted at larger scales, and studies at the regional scale are still lacking. This lack of regional-scale studies is a disadvantage for a complete and detailed understanding of the response of grassland biomass to climate change in semi-arid and arid regions. Furthermore, given that the previous studies were mostly conducted using limited field data, sufficient field-based observation data are needed to examine these results.

In this study, using field-based biomass samples (1,434 aboveground biomass samples and 64 above- and below-ground biomass samples) in the Xilingol grasslands between 2005 and 2012 and a 16-day MODIS-NDVI product with a spatial resolution of 250 m×250 m for the period 2001 to 2012, statistics-based models were established to estimate the biomass. We further examined the spatio-temporal variations in the biomass and the effects of climatic factors (temperature and precipitation) on these variations. Our purpose was to understand (1) the biomass and its changes in the Xilingol grasslands between 2001 and 2012 (2), the spatial distribution of the grassland biomass and (3) the relationships between the spatio-temporal pattern of the biomass and the climatic factors.

## Materials and Methods

### Study area

**Figure 1:**
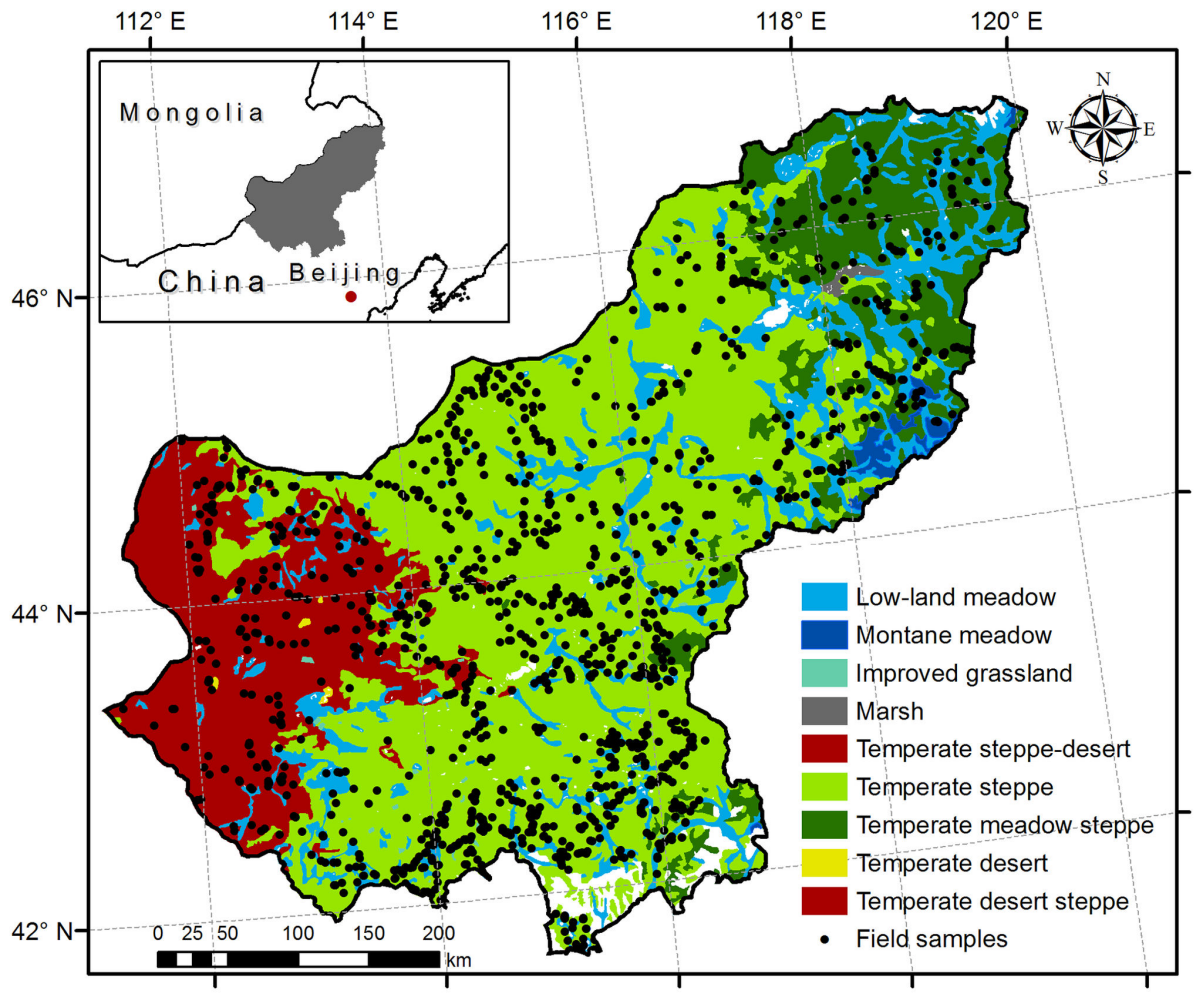
Locations of the 1,434 sampling sites across Xilingol on the background of a map of grassland types.

Xilingol is located in the central part of Inner Mongolia’s temperate grassland (111°14'~120°12′E, 41°60′~46°78′N; ca. 200,000 km^2^; Figure 1). Due to the widespread distribution of the grassland and its relatively high vegetation productivity, Xilingol plays an important role in China’s animal husbandry industry. This region has a relatively flat topography and typical vegetation communities, providing a suitable location for investigating the spatio-temporal patterns of the biomass in temperate grasslands. The area has a continental climate characteristic of the middle latitude semi-arid and arid zones. The mean annual temperature in the study area ranges from 1.3~4.8°C. Due to the effect of the Pacific monsoon, the annual precipitation exhibits a strong east-to-west gradient that decreases from more than 450 mm to less than 150 mm and occurs mainly in the summer. With this spatial distribution of the precipitation, the grassland in Xilingol is dominated by temperate meadow steppe, temperate steppe and temperate desert steppe (Figure 1). The zonal soil is classified as chernozem and chestnut (Genetic Soil Classification of China).

### In situ biomass measurements

In this study, no specific permissions were required for the grassland biomass investigation; our sampling did not involve endangered or protected species. Furthermore, the field samples were obtained from the large-scale field campaign organized by the Grassland Monitoring and Supervision Center of Ministry of Agriculture of China (GMSC), primarily in July and August from 2005 to 2012. The sampling sites, each with an area of at least 1 km^2^, were chosen to represent typical vegetation communities. For most sites, three plots (1 m×1 m) were selected, with the distance between plots being greater than 250 m. 

**Figure 2:**
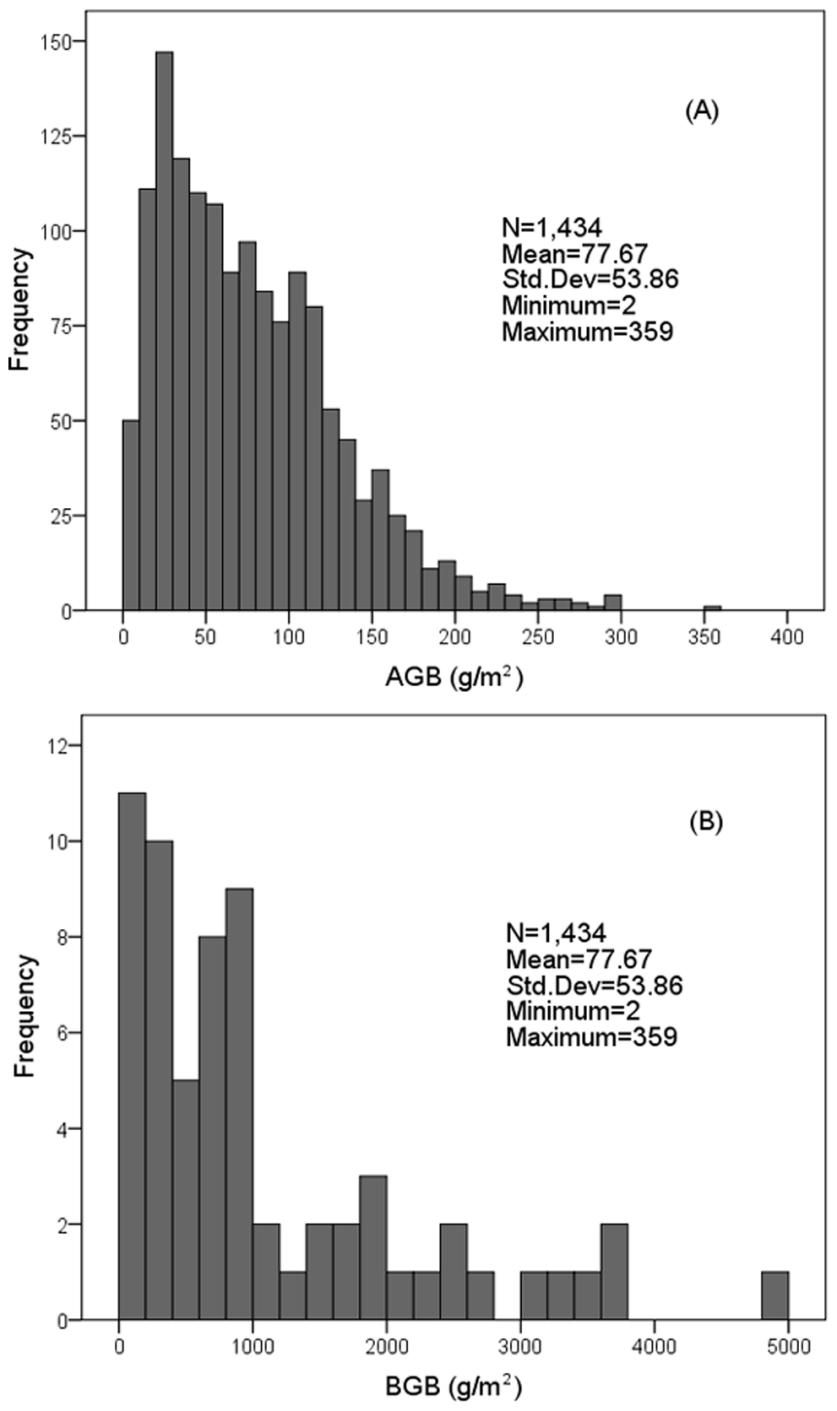
Frequency distributions of the AGB (A) and BGB (B) based on field measurements. The number of AGB–BGB samples is 9 for the temperate meadow steppe and low-land meadow (in the eastern part of Xilingol), 33 for the temperate steppe (the middle part of Xilingol) and 22 for the temperate desert steppe (the western part of Xilingol). The BGB data reported in previous publications were mostly sampled during the summers (July to September) of 2001 to 2005 (Yang et al., 2010).

To obtain the actual AGB of herbs, all above-ground plants in the three plots were harvested to measure the fresh weight. For shrubs in homogeneous grassland, one plot (10 m×10 m) was sampled. We sorted the plants (clumps) into three groups (large, medium and small) according to their size and then cut and weighed the green parts, along with the branches of the same year, of a representative plant (clump) for each group. For each plot, we multiplied the weight of the representative plant in each group by the number of plants in that group and then calculated the sum of the weights of the three groups. Because the field samples collected by the GMSC were presented in wet weight, we converted the wet weight to the air-dried weight using conversion coefficients of different grassland types [31] and further converted the air-dried weight to dry weight with 15% water content [32]. We examined and verified the sampling data and finally obtained 1,410 AGB field samples (Figure 1 and Figure 2A). 

To estimate the BGB, our group conducted two investigations across Xilingol’s mainly zonal grassland types in August of 2010 and 2012 and collected the AGB (using a measurement approach similar to that described above, but only one plot was settled for each sampling site) and the corresponding three soil cores with a diameter of 6.8 cm at depth intervals of 10 cm within each quadrat (1×1 m). The BGB were mostly collected at a depth of 60 to 80 cm, but two samples were obtained at a depth of 30 cm due to the scarcity of roots. The root samples were soaked in water and then separated from residual soil using a 0.25 mm sieve. Live roots were distinguished by their color and resilience. Finally, the biomass samples were dried in an oven at 80 °C to a constant mass. We ultimately obtained 24 above- and below-ground biomass samples across the main grassland types (Figure 2B). Additionally, we conducted a search for publications concerning biomass data in the Xilingol grasslands to supplement our actual field biomass measurements. We gathered 40 published records (AGB and BGB data, Figure 2B). These BGB records from published sources were mostly to the depth of 50 to 60 cm and sampled in the summer during 2001 to 2005 [33]. Because these published biomass data did not include the exact sampling years, we only used these records to analyze the relationship between AGB and BGB.

### Remote sensing and climate data set

MODIS data are suitable for studying grassland vegetation at a regional scale due to their spatial and temporal advantages. The MODIS data used in this study were the 16-day composite MODIS-NDVI products with a 250 m spatial resolution for the period from 2001 to 2012, available from the U. S. National Aeronautics and Space Administration (NASA). The monthly VI data were developed using the Maximum Value Composition (MVC). The NDVI of the peak season was then calculated with the average NDVI of July and August. Then, we produced the spatial distribution of the NDVI for the whole study area. Considering that NDVI data in sparsely vegetated areas are largely influenced by the spectral characteristics of the soil, we only analyzed areas with a peak season NDVI > 0.1.

The climate data, obtained from the National Meteorological Information Center (NMIC), included monthly precipitation and temperature records for 2001 to 2012 from 47 climate stations distributed across Inner Mongolia. To analyze the relationships between the biomass and climate factors, we used the Anusplin software package 4.3 to interpolate and produce a continuous spatial distribution of temperature and precipitation with a 500 m spatial resolution [34]. An error analysis of the interpolation method in our study area presented a mean relative error (REE) of 10 to 30% for monthly precipitation and a REE of < 6% for the average monthly temperature during the growing season.

### Biomass estimation

We estimated the grassland biomass in Xilingol using the following four steps. First, we calculated the mean NDVI value (in general, there are two to four pixels per mean value) within a circular area of the plot (one of three plots in a sampling site; 250 m radius) [12,35]; subsequently, we established a database of values for the site-specific actual AGB versus the NDVI of the peak season for each year. Second, we used this database to develop regression models (unitary linear, quadratic, power and exponential) for 80% of the total number of samples and their corresponding NDVI values. Third, we calculated the root mean squared error (RMSE) and the REE to evaluate the precision of the models based on the reserved samples (20% of the total samples). The RMSE and REE were calculated as follows:

$$RMSE=\sqrt{\frac{\sum {\left(Y_{i}-Y_{i}{}^{'}\right)}^{2}}{N}}$$ (1)

$$REE=\sqrt{\frac{\sum {\left[\left(Y_{i}-Y_{i}{}^{'}\right)/Y_{i}{}^{'}\right]}^{2}}{N}}$$ (2)

**Figure 3:**
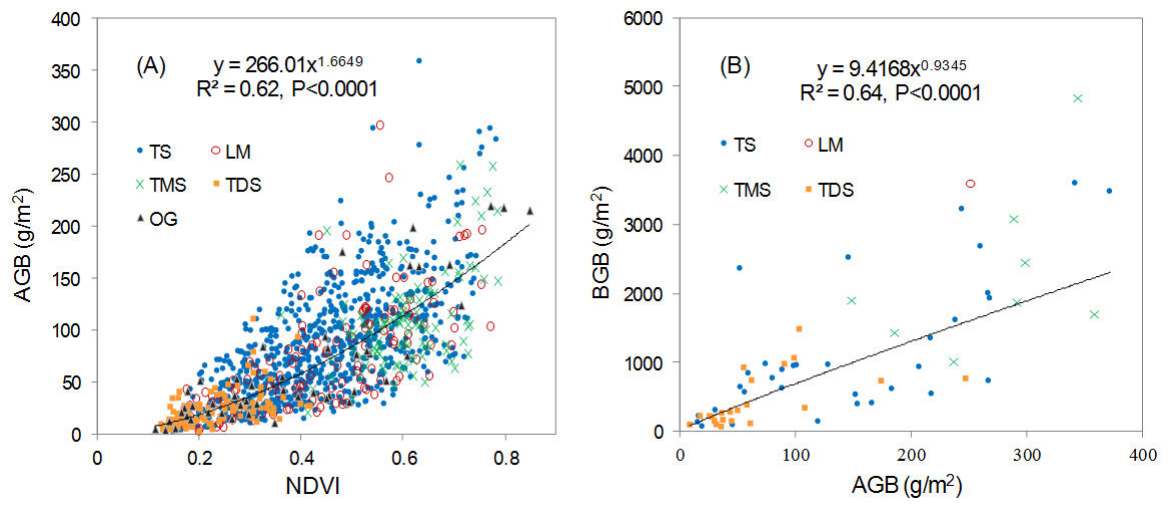
Relationships of NDVI–AGB (A) and AGB–BGB (B) for grasslands in Xilingol. TMS, temperate meadow steppe; TS, temperate steppe; TDS, temperate desert steppe; LM, low-land meadow; and OG, other grassland types, including improved grassland, temperate steppe-desert, temperate desert, montane meadow and marsh.

*Y*_*i*_ is the actual AGB (random reserved field samples), *Y*_*i*_ *'* is the estimated AGB, and *N* is the sample number. As shown in Table 1, the optimal estimating model was a power function (Figure 3A). Finally, we used the best regression model and the NDVI data to estimate the AGB for each pixel for the period 2001 to 2012 and further developed a relationship (Figure 3B) to estimate the BGB based on the estimated AGB [3,33]. MODIS-NDVI and biomass data were computed using ArcGIS 10.0 software.

**Table 1:** Statistical models between AGB and NDVI.

Model	R^2^	F value	RMSE (g/m^2^)	REE	Precision
AGB=266.01×*NDVI* ^1.6649^	0.62	1862.92	22.92	0.27	0.73
AGB=9.4514×*exp*(4.1917×*NDVI*)	0.57	1511.10	26.03	0.29	0.71
AGB=100.98×*NDVI* ^2^+161.2×*NDVI*−12.633	0.51	595.72	25.12	0.29	0.71
AGB=249.44×*NDVI*−29.407	0.51	1181.05	25.61	0.30	0.70

Note: The numbers of regress/test samples are 1,147 and 287, respectively; P<0.0001; precision=1-REE.

### Analysis of relationships between AGB and climatic factors

The green-up data are from approximately early May in China’s northern temperate grassland [36,37], and the peak standing AGB was investigated in this study. Thus, we generated the main growing season (May to August) mean temperature (GST) and the main growing season precipitation (GSP). To examine the climatic factors affecting the inter-annual variations of the biomass, we analyzed the relationships between the inter-annual biomass anomaly index (BAI) and growing season temperature anomaly index (TAI) and between the BAI and growing season precipitation anomaly index (PAI). Because the BGB may accumulate for several years, we only analyzed the relationships between the AGB and climatic variables. The BAI was calculated according to the following equation:

$$BAI=(AGB_{i}-\bar{AGB})/\bar{AGB}$$ (3)

*AGB*_*i*_ is the AGB of an individual year in Inner Mongolia, and $\bar{AGB}$ is the mean AGB during 2001~2012. PAI and TAI were calculated using the same method.

To describe the spatial variations in the AGB and explore their climatic controls, we averaged the values of AGB in 2001~2012 for each pixel-based estimation by remote sensing. GSP and GST for each pixel were also calculated by averaging the climatic data from 2001 to 2012, which were interpolated by Anusplin. Additionally, we also analyzed the relationship between the AGB and the moisture index K, developed especially for Chinese grasslands [38,39]. We improved this moisture index K to examine the combined influence of the GSP and GST in this study. The *K* was calculated according to the followed equation: 

K=GSP/(AGST×0.1) (4)

*GSP* is the growing season precipitation, and *AGST* is the growing season accumulated temperature. All statistical analyses were performed using SPSS 17.0 software.

## Results

### Interannual changes in biomass

Using the two functions (Figure 3) and the NDVI time series data, we calculated the biomass in the Xilingol grasslands over the 12-year period. The total biomass was estimated to be 99.05 Tg (1 Tg=10^12^g) in a total area of 19.6×10^4^ km^2^, with 12.28 Tg and 86.78 Tg occurring in the AGB and BGB, respectively (averaged over the 12 years). During 2001 to 2012, the biomass in the study area experienced a general fluctuation (the coefficient of variance for the yearly biomass was 19.26%), with no significant trend (R^2^=0.02, P=0.66). All of the main grassland types exhibited similar trends (Table 2).

**Table 2:** Biomass densities of the main grassland types in Xilingol.

Grassland type	Area (10^4^ km^2^)	AGB density (g/m^2^)								AGB (Tg)			
		2001~2003		2004~2006	2007~2009			2010~2012		2001~2003	2004~2006	2007~2009	2010~2012
TMS	2.5	113.2		119.3	96.1			116.5		2.8	3.0	2.4	2.9
TS	10.8	60.7		59.9	51.6			66.2		6.5	6.5	5.6	7.1
TDS	2.9	20.5		18.5	22.0			23.7		0.6	0.5	0.6	0.7
LM	2.6	79.8		83.2	67.4			81.7		2.1	2.2	1.8	2.1
OG	0.8	55.5		55.4	47.8			55.2		0.4	0.4	0.4	0.4
Total	19.6	63.7		64.1	54.7			67.8		12.5	12.6	10.7	13.3
		BGB density (g/m^2^)								BGB (Tg)			
TMS	2.5	779.5	819.2			667.9	801.4			19.4	20.4	16.6	20.0
TS	10.8	431.8	427.0			371.3	468.8			46.6	46.0	40.0	50.6
TDS	2.9	157.1	142.9			168.1	179.5			4.6	4.2	5.0	5.3
LM	2.6	557.1	579.2			475.0	570.6			14.5	15.1	12.4	14.9
OG	0.8	388.4	386.1			338.6	387.3			3.0	3.0	2.6	3.0
Total	19.6	449.7	452.7			390.9	477.8			88.1	88.7	76.6	93.7
		TB density (g/m^2^)								TB (Tg)			
TMS	2.5	892.7	938.5			764.0	917.9			22.2	23.4	19.0	22.9
TS	10.8	492.5	486.8			422.9	535.0			53.1	52.5	45.6	57.7
TDS	2.9	177.6	161.4			190.1	203.2			5.2	4.8	5.6	6.0
LM	2.6	636.9	662.4			542.3	652.3			16.6	17.3	14.1	17.0
OG	0.8	444.0	441.6			386.4	442.5			3.4	3.4	3.0	3.4
Total	19.6	513.4	516.9			445.6	545.6			100.5	101.3	87.4	107.0

Note: TB, total biomass; TMS, temperate meadow steppe; TS, temperate steppe; TDS, temperate desert steppe; LM, low-land meadow; and OG, other grassland types, including improved grassland, temperate steppe-desert, temperate desert, montane meadow and marsh.

### Spatial distribution of biomass

**Figure 4:**
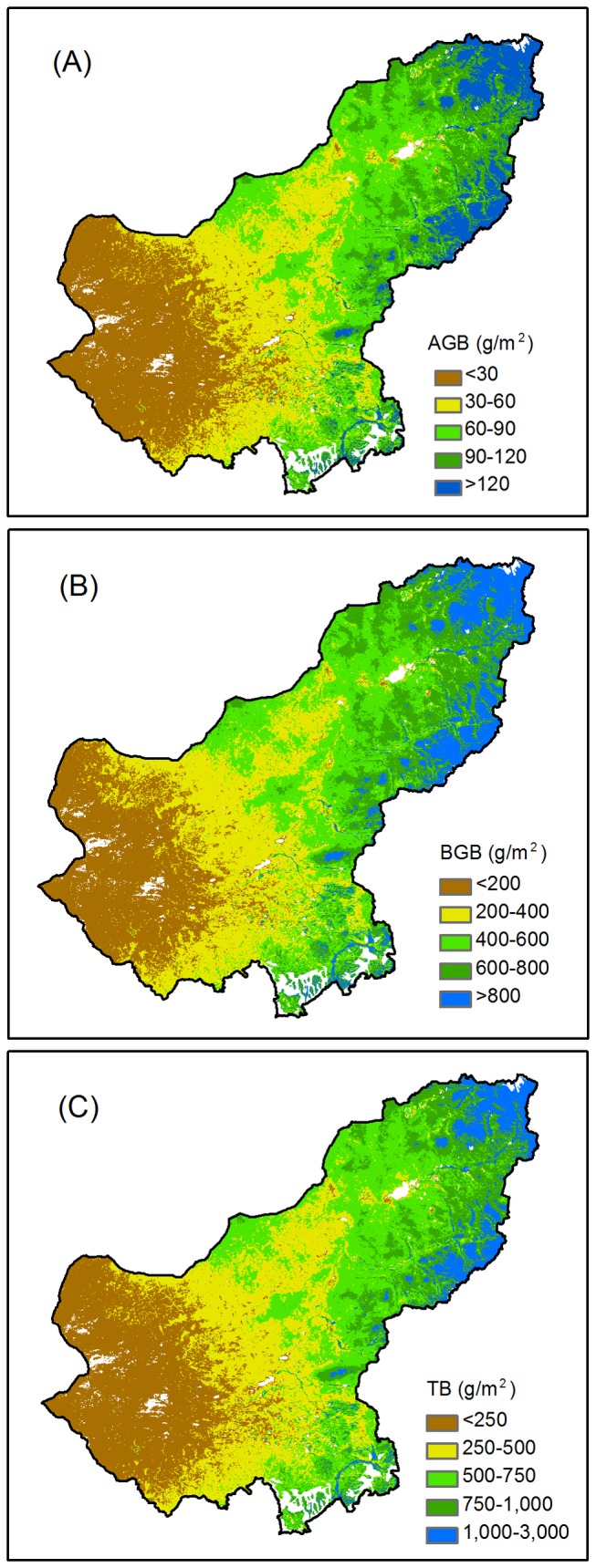
The 12-year-averaged biomass density between 2001 and 2012 in the Xilingol grasslands.

Our results demonstrated that the biomass in the Xilingol grasslands exhibits a large spatial difference and gradually decreases from northeast to southwest (Figure 4). The mean biomass density was 505.4 g/m^2^, with 62.6 g/m^2^ in the AGB and 442.8 g/m^2^ in the BGB over the entire study area during the past 12 years (average of the 12 years, Table 2). A high-density biomass (>750 g/m^2^) appeared in the northeastern part in the distributed temperate meadow steppe and belt-shaped low-land meadow near Dahingganling Mountain. A medium biomass density appeared mostly in the middle part of Xilingol, where the temperate steppe is primarily distributed (250 to 750 g/m^2^). The biomass in the western part of Xilingol, where the central region of the Otindag Sandland is located and where the temperate desert steppe is primarily distributed, has the lowest density (<250 g/m^2^).

### The relationship between the AGB and climate factors

**Figure 5:**
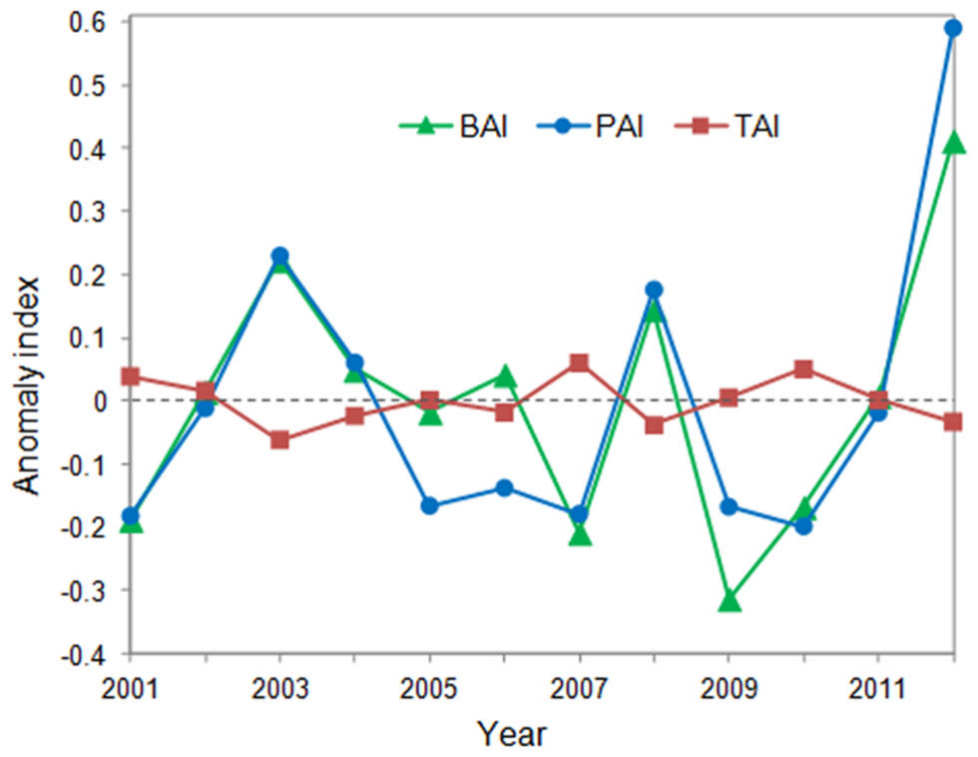
Coupled patterns between the AGB and climate factors (GSP and GST). BAI: above-ground biomass anomaly index; PAI: growing season precipitation anomaly index; and TAI: growing season temperature anomaly index.

We analyzed the relationships between AGB and climate variables to explore the response of the AGB to climate change. In the period from 2001 to 2012, both the GST and GSP in the research area exhibited fluctuations, and there was no significant trend in the variations (P>0.05). Figure 5 indicates that during the period from 2001 to 2012, the inter-annual dynamic in the AGB was generally consistent with the variation in the GSP and exhibited a robust positive correlation (R^2^=0.82, P<0.001). In contrast, we found that the relationship between the AGB and GST exhibited an opposite pattern with the AGB compared with the GSP (Figure 5), showing a negative correlation (R^2^=0.61, P=0.003).

**Figure 6:**
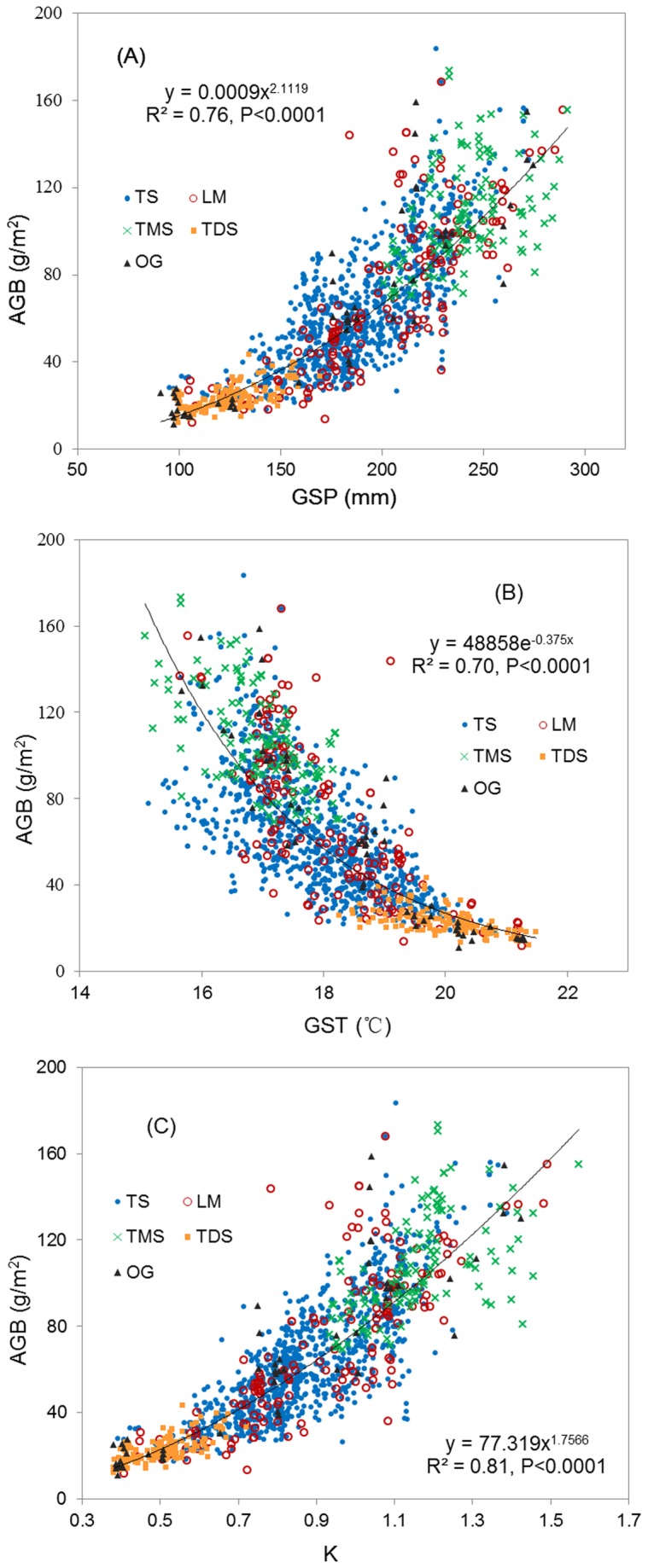
Relationships between the AGB and climate factors. (A): Growing season precipitation (GSP); (B): growing season temperature (GST); and (C): moisture index (K). Each data point in the figure represents the 12-year average value for the period from 2001 to 2012. TMS, temperate meadow steppe; TS, temperate steppe; TDS, temperate desert steppe; LM, low-land meadow; and OG, other grassland types, including improved grassland, temperate steppe-desert, temperate desert, montane meadow and marsh.

We averaged the values of the AGB estimated by remote sensing from 2001 to 2012 for 1,434 field-measured locations and analyzed the relationships between the AGB and climatic factors. The results indicated that the variance of the AGB was strongly related to the GSP and GST (Figure 6). The AGB increased progressively with the precipitation gradient (Figure 6A), and their relationship could be fit by a power function (R^2^=0.76, F=4535, P<0.0001) or an exponential function (R^2^=0.76, F=4525, P<0.0001). Although a linear function could also be used to fit the correlation, the R^2^ and F values were lower (R^2^=0.70, F=3256, P<0.0001). However, the AGB decreased exponentially as the GST increased (Figure 6B; R^2^=0.70, F=3274, P<0.0001). We also analyzed the relationship between the AGB and a moisture index (K) that takes into account both the GSP and GST and found that the K could explain more spatial variance of the AGB than the GSP alone (Figure 6C; R^2^=0.81, F=6098, P<0.0001).

## Discussion

### Estimate of the biomass in the Xilingol grasslands

In this study, the grassland biomass densities were estimated to be 878.3 g/m^2^ for the temperate meadow steppe, 484.3 g/m^2^ for the temperate steppe and 183.1 g/m^2^ for the temperate desert steppe, which are lower than previous estimates. We used the temperate steppe, which consists primarily of the spread grassland type, as an example to illustrate differences among the estimates. Ma et al. [10] gathered 113 field samples from Inner Mongolia’s grasslands and determined that the biomass density was 822.3 g/m^2^ for the temperate steppe. By using the NOAA-NDVI and field-based biomass data, Piao et al.[11] and Ma et al. [3] estimated the biomass density of the temperate steppe in China to be 710.2 g/m^2^ and 779.1 g/m^2^, respectively (a carbon conversion factor of 0.45 was used to compare the estimates in these studies) [32]. The differences among these estimates may result from two key factors. First, the estimates may have been dramatically affected by field biomass measurements. For example, Ma et al. [3] investigated the biomass in northern China’s grasslands using AVHRR-NDVI and field-based samples. Their AGB samples were collected in enclosed or ungrazed grasslands, showing an average value of 136.2 g/m^2^ (based on 41 samples) for the temperate steppe of Inner Mongolia, which is approximately 50 g/m^2^ higher than of the value obtained in our study (83.5 g/m^2^, based on 930 samples). Fan et al. [9] harvested litter biomass in addition to standing AGB; their samples for temperate steppe showed an average value of 151.1 g/m^2^ (based on 113 samples). The field samples examined in this study were found in typical areas, and only standing AGB was collected. The different plot locations and harvest targets employed in these studies may have contributed to such differences [16]. On the other hand, the different approaches applied, including BGB estimations, were a critical factor. For example, Ma et al. [10] calculated the biomass densities for different grassland types based on field samples and further estimated the biomass according to different grassland types. Compared with this method, the satellite-based approach can represent the spatial details of the AGB across the entire study area, thereby reducing the estimation uncertainty [8].

### Effects of climatic factors on interannual changes in AGB

Our results indicate that the biomass in Xilingol’s grassland has experienced a general fluctuation, which is affected by climatic inter-annual variation. These results demonstrate a close coupling between the AGB and GSP (R^2^=0.82, P<0.001), indicating that precipitation is a primary determinant for the inter-annual pattern of the AGB in temperate grasslands. This result was consistent with the most previous studies, but the correlation in our study is more robust [3,18,40]. We inferred that the high sensitivity to changes in precipitation in Xilingol’s temperate grassland is a critical reason for the strong positive correlation. In contrast, a negative correlation was found between the GST and AGB, though with a lower significance (R^2^=0.61, P=0.003). This result did not support the findings of previous studies. For example, Piao et al. [11] suggested that an increase in temperature may promote vegetation productivity in China’s grasslands. Ma et al. [3] found that biomass in an alpine meadow was positively correlated with temperature. Differences in the studied regions may be a key factor responsible for these contrasting results. In the temperate grassland in Xilingol, precipitation is the primary growth-limiting factor, although rising temperatures may promote water evaporation and further induce an intensified drought. In contrast, in the alpine meadow, low temperatures were most likely an important factor constraining plant growth. These distinct impacts of climatic factors on biomass among different grassland types may be due to the different composition of plant functional types [28]. Therefore, we conclude that the impacts of climatic factors on inter-annual patterns of grassland biomass are regionally diverse and that these variations may be ignored at larger scales. Multi-scale studies are needed to improve our understanding of how grasslands respond to climate change and the underlying mechanisms. In addition, we found a negative correlation between the inter-annual GSP and GST (R^2^=0.50, P=0.01), which also contributed to the inter-annual patterns observed for GST–AGB. 

### Effects of climatic factors on spatial variation in AGB

A strong positive GSP–AGB relationship was found in the Xilingol grasslands, which is similar to previous work [10,19,23,27,41]. We found that both power and exponential functions could be used to fit the relationship, partly supporting the previous results [24,28]. In previous studies, linear and exponential relationships have been reported. Hu et al. [24] inferred that if samples are sufficient, the slope of the precipitation–ANPP relationship may increase as the climate shifts from arid to humid. This gradual change in the slope may be caused by the difference in the water use among various grassland types across the precipitation gradient [28]. In the present study, we also found the various slopes for the relationship, but they were not as steep as the function reported by Guo et al. [28]. We inferred that this disagreement may be due to the difference in the study scale between the two studies. Guo et al.’s study [28] area was larger than ours, and their precipitation gradient was greater than that in the present study. Thus, the additional plant communities induced the greater slope difference in the precipitation–AGB relationships. Our finding supports the assumption that vegetation–climatic factor relationships are scale dependent [30].

**Figure 7:**
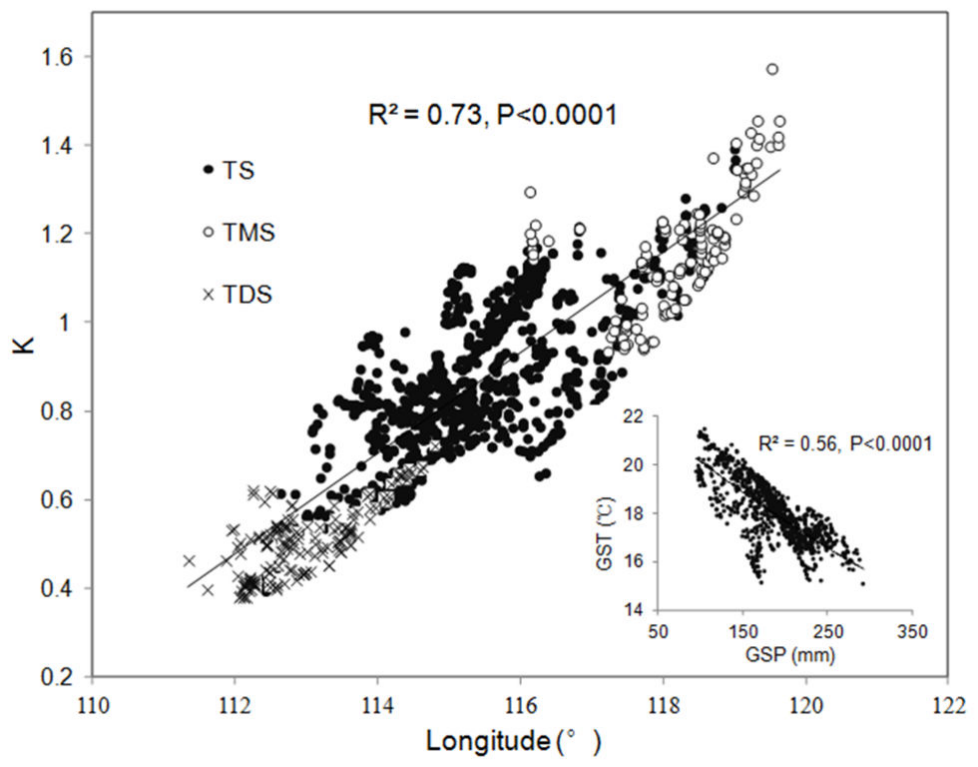
The western-eastern spatial distribution of the moisture index (K) and the relationship between the GSP and GST in Xilingol. Each data point in the figure represents the 12-year averaged value for the period from 2001 to 2012 (n=1218). TMS, temperate meadow steppe; TS, temperate steppe; and TDS, temperate desert steppe.

We also found a significant negative relationship between the GST and AGB. This result may be due to the effect of the GST and GSP interactions on the vegetation growth [42]. High temperatures promote water loss and further constrain plant growth in dry grasslands. In addition, the GST increases as the GSP decreases from the eastern part to the western part in Xilingol (R^2^=0.56, P<0.0001), and the hydro-thermal distribution is characterized by warm and dry conditions in the western temperate desert steppe to cold and wet conditions in the eastern temperate meadow steppe (Figure 7). This spatial pattern was also an important factor in the negative correlation. Furthermore, we inferred that this GST–GSP pattern may dramatically impact the slope of the GSP–AGB relationships along the GSP gradient. Figure 7 describes the western-eastern spatial distribution of the moisture index (K) and the relationship between the GSP and GST. In the western temperate desert steppe, the lack of available water is due to not only the low GSP but also to the high evaporation caused by a high GST. However, the opposite situation appeared in the eastern temperate meadow steppe. Therefore, this coupled GST–GSP effect should also be considered to explain the underlying mechanisms of the increase in the slope of the precipitation–AGB relationship across the precipitation gradient.

## Conclusions

Spatio-temporal patterns of biomass and their relationships with climate factors were investigated in the Xilingol grasslands. In general, our results suggest that grassland biomass fluctuated between 2001 and 2012 and showed a spatial distribution with a decreasing trend from east to west. The interannual variation of AGB was mainly driven by GSP. We also observed a power function of GSP on the spatial variation of AGB and further inferred that the coupled effects of GST and GSP may have been a critical factor causing the increasing slope of the GSP–AGB relationship. Our findings indicate that the impacts of climatic factors on the spatio-temporal patterns of grassland biomass are regionally diverse, which partially supports the idea that the observed vegetation–climatic factor relationships may be scale dependent. Considering the various types of grasslands and the spatial-temporal heterogeneity of climate change, multi-scale studies and long-term field data are necessary to examine the relationships between biomass and climatic factors. This study provides insight into approaches for understanding how grasslands respond to climate change.
